# Visfatin Connection: Present and Future in Osteoarthritis and Osteoporosis

**DOI:** 10.3390/jcm8081178

**Published:** 2019-08-07

**Authors:** Eloi Franco-Trepat, María Guillán-Fresco, Ana Alonso-Pérez, Alberto Jorge-Mora, Vera Francisco, Oreste Gualillo, Rodolfo Gómez

**Affiliations:** 1 Musculoskeletal Pathology Group, Institute IDIS, Santiago University Clinical Hospital, SERGAS, 15706 Santiago de Compostela, Spain; 2 Research laboratory 9, Institute IDIS, Santiago University Clinical Hospital, SERGAS, 15706 Santiago de Compostela, Spain

**Keywords:** osteoarthritis, osteoporosis, cartilage, bone, inflammation, catabolism, visfatin, NAMPT, TLR4, metabolic alterations

## Abstract

Musculoskeletal pathologies (MSPs) such as osteoarthritis (OA) and osteoporosis (OP), are a set of disorders that cause severe pain, motion difficulties, and even permanent disability. In developed countries, the current incidence of MSPs reaches about one in four adults and keeps escalating as a consequence of aging and sedentarism. Interestingly, OA and OP have been closely related to similar risk factors, including aging, metabolic alterations, and inflammation. Visfatin, an adipokine with an inflammatory and catabolic profile, has been associated with several OA and OP metabolic risk factors, such as obesity, insulin resistance, and type II diabetes. Furthermore, visfatin has been associated with the innate immune receptor toll-like receptor 4 (TLR4), which plays a key role in cartilage and bone inflammatory and catabolic responses. Moreover, visfatin has been related to several OA and OP pathologic features. The aim of this work is to bring together basic and clinical data regarding the common role of visfatin in these pathologies and their major shared risk factors. Finally, we discuss the pitfalls of visfatin as a potential biomarker and therapeutic target in both pathologies.

## 1. Introduction

Musculoskeletal pathologies (MSP) are a set of disorders that cause severe pain, motion difficulties and even permanent disability. Currently, the incidence and prevalence of these diseases reaches about 1 in 4 adults in developed countries, as a consequence of aging and unhealthy sedentary lifestyles [1,2,3]. The future is even more unsettling, and estimations foresee restless escalation [3]. Interestingly, aging and metabolic alterations are common features of osteoarthritis (OA) and osteoporosis (OP), two major MSPs that share distorted levels of similar inflammatory, catabolic and metabolic factors [4,5,6,7].

Altogether, this common catabolic and inflammatory environment contributes to the onset of both pathologies in the cartilage and bone. In agreement with this, OP development was also related to OA-like cartilage alterations [8]. Interestingly, visfatin, an adipokine involved in inflammation and catabolism [9,10,11], has been strongly associated to several pathological features and risks factors of OA and OP, like obesity [9,10,12,13,14,15], and diabetes mellitus [16,17,18]. As such, visfatin might prove to be a common denominator of OA and OP.

In this review, we describe the role of visfatin in the pathological context of OA and OP, as well as its relationship with shared risk factors between both pathologies. Finally, we depict the potential role of visfatin as a biomarker for both diseases.

## 2. Essentials: OA, OP, and Visfatin

### 2.1. Osteoarthritis (OA)

**Figure 1:**
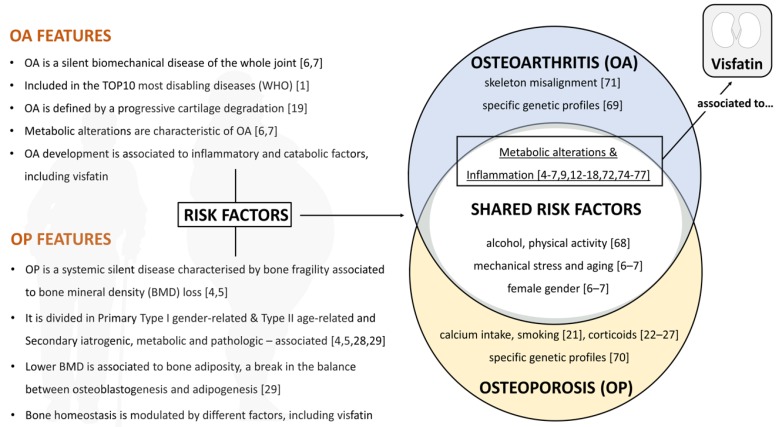
Osteoarthritis (OA) and osteoporosis (OP). OA and OP are two major silent rheumatic diseases with specific features, and are included in the World Health Organization (WHO) disease-disabling lists. Nevertheless, they clearly present different clinical diagnoses and development. OA is highly associated to cartilage degradation and OP is associated to bone mineral density (BMD) alterations. Interestingly, both diseases not only manifest unique risk factors, but also shared ones. Among the shared risk factors stand inflammation and metabolic alterations. Interestingly, both shared risk factors are associated to visfatin, a life-essential adipokine.

Osteoarthritis (OA) is eminently a biomechanical disease, but its development and onset are strongly associated with inflammatory and catabolic alterations [6,7]. In fact, both skeleton misalignments and metabolic factors, among others, contribute to the progressive degradation of the articular cartilage and the characteristic joint space narrowing. Eventually, the cartilage wears away and prompts a bone-to-bone abrasive articulation that causes severe pain, stiffness, and disability [19]. OA is considered a disease of the whole joint as it also affects tendon, periarticular muscles, and synovium, just not the subchondral bone and articular cartilage [19]. The slow and silent progression of the disease impedes a premature diagnosis, and it is usually noticed at the late stages, when preventive measures no longer work [6,7,20] (Figure 1).

As a result, the World Health Organization have included OA as one of the ten most disabling diseases in developed countries [1]. OA development and progression has been linked to risk factors such as joint mechanical stress, metabolic disorders, female sex, some genetic profiles, and aging, but also to inflammation [6,7] (Figure 1). In fact, it is known that the altered permeability of the synovium in OA facilitates the entrance of plasma proteins into the synovial fluid, and they are responsible for activating the innate immune receptor toll-like receptor 4 (TLR4) [7].

### 2.2. Osteoporosis (OP)

Osteoporosis (OP) is a systemic disease characterized by bone fragility, associated with a dramatic loss of bone density [4,5]. These bone alterations are increased during aging, and increase the likeliness of bone fractures [5]. As a matter of fact, the post-menopausal stage and amenorrhea are two aged-related risk factors that contribute to the increased incidence of OP bone fractures in women [5]. Other risk factors include the absence of physical activity, low calcium intake, excessive alcohol consumption, smoking [21], and corticoid treatment [22,23,24,25,26,27].

OP is a silent disease that progresses undetected for many years without symptoms until a fracture occurs. Considering the multifactorial etiology of this chronic pathology, it has been classified into primary and secondary. Moreover, primary osteoporosis comprises both gender-related type I OP and age-related type II osteoporosis [5]. As such, type I OP is linked to the reduction of sex hormone levels in post-menopausal women, and type II OP is associated with systemic senescence and reduced stem cell precursors [5]. Secondary osteoporosis includes iatrogenic OP [28], metabolic OP [29], and other pathology-associated OPs [5]—which are connected to other specific etiologic mechanisms. Osteoporosis is diagnosed by a bone mineral density (BMD) test, which is a painless method to detect bone density alterations. A drastic reduction of BMD is common in both primary and secondary OP [4,5,29]. Lower BMD has been associated with bone adiposity, which is a consequence of the unbalance between osteoblastogenesis and adipogenesis at the bone marrow of OP patients [29] (Figure 1).

Despite having clearly different clinical diagnoses and development, many rheumatic diseases, including OA and OP, manifest similar characteristics across diverse musculoskeletal tissues [30]. Consistent with this, it is noteworthy that in vivo models of OP development were associated with an increased OA severity and the appearance of OA-like alterations in the cartilage [8].

### 2.3. Visfatin (NAMPT/PBEF)

The adipokine visfatin, formerly known as the pre-B-colony enhancing factor (PBEF) [31], is broadly and differently expressed in multiple tissues [32], including those from the musculoskeletal system (muscle, bone, synovium, and cartilage) [31,32,33,34,35,36]. Nevertheless, the adipose tissue, including visceral and subcutaneous fat, is the most important visfatin source [12]. Interestingly, visfatin secretion by the visceral adipose tissue is higher than the subcutaneous fat [9,12]. This nearly ubiquitous expression of visfatin suggests a key biological role for this adipokine; in fact, its homozygous deletion in mice is lethal [37]. Confirming this key biological role, the homology of its protein sequence among other mammals is above 94% [38,39].

**Figure 2:**
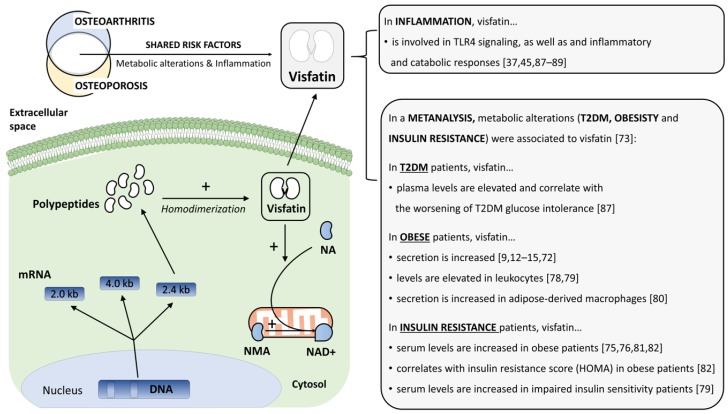
**Visfatin** Interaction with Osteoarthritis (OA) and Osteoporosis (OP) Risk Factors. Intracellular visfatin synthesis and its enzymatic activity. Intracellular visfatin synthesis and its enzymatic activity. Among the shared risk factors between OA and OP, stand inflammation, and several metabolic alterations (type 2 diabetes mellitus (T2DM), obesity, and insulin resistance). Interestingly, both risk factors are associated to visfatin, a life-essential enzyme. Nicotinamide (NA); nicotinamide mononucleotide adenyltransferase (NMA); nicotinamide adenine dinucleotide (NAD+).

Visfatin is a class type II phosphoribosyl transferase homodimer [40,41] of about 120 kDa [42,43,44]. The two 473-residue polypeptides of 52 kDa each are encoded from the 2.4 kb mRNA, whereas the roles of 2.0 kb and 4.0 kb transcripts are unknown. Transcription from the human visfatin gene (7q22; 34.7 kb) is regulated by two different promoters and modulated by alternative splicing [31,32] (Figure 2).

At the cellular level, visfatin is secreted into the extracellular space [45,46] by an unclear mechanism [45,46]. Although subcellular distribution is still under debate [38], visfatin has been identified in the nuclei [47,48] associated to cell cycle regulation [38], and at the cytosol [49] associated to its enzymatic activity as nicotinamide phosphoribosyl transferase (NAMPT) [36]. This activity is linked to the generation of NAD^+^ [50], a fundamental energy and signaling molecule found across the majority of organisms [51,52], both eukaryote and prokaryote [53,54].

The mammalian NAD^+^ salvaging pathway starts in the cytosol with nicotinamide (NA) as the substrate and visfatin as the essential limiting enzyme whose expression is critical [36,42,50,55,56]. After this bottleneck, the pathway continues in the mitochondria with the nicotinamide mononucleotide adenyltransferase (NMA) and the synthesized NAD^+^ [36,41,42,50,55,56,57,58,59]. As a result, any NAD^+^ dependent process is bound to be regulated by visfatin, and this includes cell adhesion [60], redox potential [61], and oxidative stress [62,63]; but also aging [64,65,66,67] via DNA repair [66], and longevity by sirtuins modulation [67] (Figure 2).

## 3. Visfatin in OA & OP Shared Risk Factors

### 3.1. The Metabolic Component

OA and OP are two widely different pathologies that share multiple risk factors, such as the absence of physical activity [68], excessive alcohol consumption, female gender, mechanical stress, inflammation, and aging [6,7]. Nevertheless, there are unique genetic profiles for OA [69] and OP [70]. Moreover, skeleton misalignment [71] is a specific OA risk factor, whereas low calcium intake [72], smoking [21] and corticoids [22,23,24,25,26,27] are OP-specific (Figure 2).

It is noteworthy that OA and OP have been associated with several metabolic alterations, most of them part of metabolic syndrome [4,5,6,7]. Interestingly, visfatin was also associated to several OA and OP shared metabolic alterations, namely cardiovascular hypertension and obesity [9,12,13,14,15,73,74], insulin resistance [75,76], and type 1 (T1DM) [16] and 2 diabetes mellitus (T2DM) [17,18,77] (Figure 2).

Specifically, obese patients have elevated secretion levels of visfatin [9,12,13,14,15,73,74]. Moreover, leukocytes’ visfatin levels are higher in those patients compared to lean ones [78,79], which correlates with an increased visfatin secretion by adipose tissue-derived macrophages [80]. Insulin resistance is a major component of the obesity pathophysiology, and consequentially, the association with visfatin is generally found, but not exclusively, in studies about obesity [75,76,81] (Figure 2). In fact, visfatin serum levels are increased in obese patients and correlate with the homeostasis model assessment of insulin resistance (HOMA-IR) [82]. Besides, visfatin serum levels were found to be higher in impaired-insulin-sensitivity patients [79] (Figure 2). Although some reports did not detect a correlation between visfatin and insulin resistance [83,84], those discrepancies might be explained by the differences in data normalization [85], and gender differences [15].

Interestingly, T2DM, a risk factor for OA and OP, has also been linked to visfatin. T2DM patients have significantly higher plasma levels of visfatin compared to healthy subjects, even after adjustment for body mass index [18]. Some studies have linked even higher plasma levels of visfatin with the worsening of T2DM glucose intolerance [86]. Recently, catabolic and inflammatory responses related to T2DM-associated OA have been shown to be mediated by TLR4 [87]. Consistent with this, visfatin has been involved in both T2DM and TLR4 signaling [37,45,87,88,89] (Figure 2).

Altogether, visfatin has shown to be a critical element involved in all major OA and OP risk factors and comorbidities. In fact, an extensive metanalysis further confirmed the association of visfatin with metabolic syndrome, cardiovascular diseases, obesity, insulin resistance and T2DM [76].

### 3.2. The Inflammatory Component

It has been widely described that inflammation promotes catabolic and degradative processes in cartilage and bone that affect their normal function [6,7,90,91,92]. As a result, inflammation is considered a risk factor for the development and perpetuation of certain types of OA and OP [6,7]. In fact, the innate immune receptor TLR4 has been linked to OA and OP development [6,90,93]. Specifically, this receptor recognizes conserved structures of pathogens, called pathogen-associated molecular patterns (PAMPs), but also damage-associated molecular patterns (DAMPS) from damaged tissues, including those associated to degenerative pathologies, such as OA [6]. Nonetheless, other inflammatory factors and mediators, such as diverse cytokines, have also been involved in the development of those diseases [6,7,90,91,92].

According to the links between OA and OP to these inflammatory responses, it is noteworthy that visfatin also participates in the promotion of certain inflammatory processes [9,10,11]. Although visfatin has been controversially related with insulin receptor [12,94], the specific receptor for visfatin has yet to be described. Nonetheless, it was recently described that visfatin directly binds to the TLR4 receptor and activates an inflammatory response in pulmonary cells [88,89], which goes in accordance with other, prior reports [37,45]. Consistent with this, liquid biopsy analysis from patients with different backgrounds found visfatin serum levels positively correlated with enhanced expression of pro-inflammatory factors [10,15,35,95], such as IL6, tumor necrosis factor α (TNFα), and C-reactive protein (CRP) [35]. Supporting this correlation, interleukin 1β (IL1β), TNFα, and IL6 were induced by visfatin in human leucocytes and monocytes [35]. Furthermore, in vivo models confirmed that inflammatory environments were associated with higher circulating levels of visfatin [35,37]. In fact, it was also described in mice that visfatin administration induced IL6 circulating levels [35]. Nonetheless, the link of visfatin and inflammation was further underpinned by the ability of visfatin and key inflammatory factors like IL6, IL1β, TNFα, and TLR4 agonists to form a positive feedback loop [32,35,96,97,98].

## 4. Visfatin Role in Osteoarthritis

### 4.1. OA and Visfatin Connection

**Figure 3:**
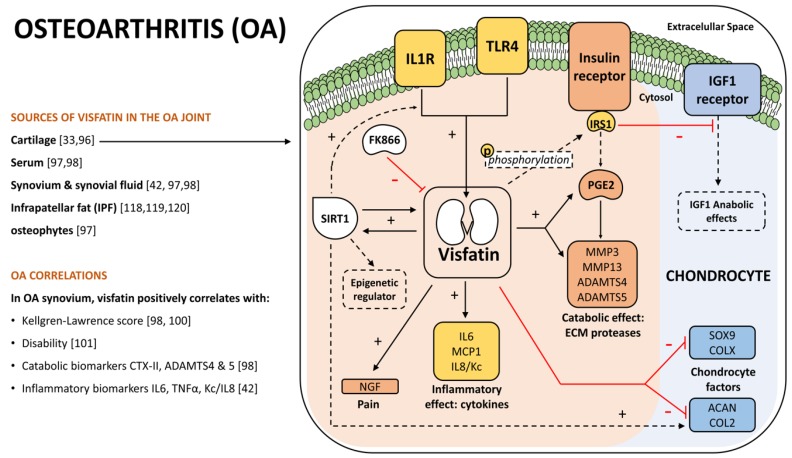
Visfatin Role in Osteoarthritis (OA). Visfatin is involved in OA. Visfatin positively correlates with several disease markers in the OA synovium. In the OA chondrocyte, visfatin expression is promoted by interleukin-1 receptor (IL1R) and innate immune toll-like receptor 4 (TLR4) and blocked by the specific inhibitor FK866. Visfatin promotes inflammatory effects, inducing the expression of cytokines, metalloproteinases, and synthesis of prostaglandin E2 (PGE2). Visfatin also works as an epigenetic regulator through its interaction with Sirtuin 1 (SIRT1). Furthermore, visfatin inhibits chondrocyte anabolism. Interleukin 6 (IL6); Interleukin 8 (IL8), also known as chemokine (Kc); monocyte chemoattractant protein 1 (MCP1); insulin receptor (IR); insulin receptor substrate 1 (IRS1); insulin-like growth factor (IGF1); extracellular matrix (ECM); matrix metalloproteinases 3 (MMP3) and 13 (MMP13); disintegrin and metalloproteinases with thrombospondin motifs 4 (ADAMTS4) and 5 (ADAMTS5); SRY-box 9 (SOX9); collagen type 10 (COLX) and type 2 (COL2); aggrecan (ACAN); nerve growth factor (NGF).

In agreement with the OA pathophysiology, multiple reports support the involvement of visfatin in the disease [33,99]. Baseline levels of visfatin, in serum and synovial fluid particularly, were especially increased in OA patients when compared to healthy controls [100,101]. Interestingly, the concentration of visfatin was higher in the synovial fluid of OA patients than in their serum-paired samples [100]. Additionally, despite certain reports [102], visfatin expression was also increased in OA cartilage in comparison to normal cartilage [33,99]. Furthermore, visfatin expression in the synovium of OA patients correlated with cartilage degradation biomarkers C-terminal telopeptide of type II collagen (CTX-II) [101]; a disintegrin; and metalloproteinases with thrombospondin motifs 4 (ADAMTS4) and 5 (ADAMTS5) [101]; as well as with Kellgren-Lawrence score [101,103]; and disability [104] (Figure 3). According to that, a catabolic role for visfatin was suggested in the OA-joint [101]. Underpinning those data, the intraarticular injection of adenovirus expressing visfatin, induced OA in mice, was described [105].

### 4.2. OA Catabolism and Inflammation

There are several works supporting the pro-catabolic activity of visfatin in the OA-joint. Studies performed in human articular chondrocytes showed that visfatin blocks the anabolic actions of the insulin-like growth factor 1 (IGF1) [106], a key factor involved in cartilage homeostasis [107]. This catabolic effect of visfatin was mediated by its ability to phosphorylate the insulin receptor substrate 1 (IRS1), which inhibited IGF1 signaling [106]. In addition, visfatin was described as inducing the synthesis of prostaglandin E2 (PGE2) in human OA and mouse chondrocytes [33,108]. The induction of PGE2, a well-known cartilage catabolic factor, was mediated by the ability of visfatin to activate the downstream signaling pathway of the insulin receptor [108]. Interestingly, visfatin was found necessary for IL1β-mediated PGE2 synthesis in mice [33]. Moreover, IL1β stimulation in human OA [33] or rabbit chondrocytes [96] induced visfatin expression. Altogether, these studies suggested a key role of visfatin in PGE2 regulation [33] (Figure 3).

The importance of visfatin in the downstream catabolic effects of IL1β was further supported by the ability of visfatin to block dedifferentiation in IL1B-stimulated chondrocytes [96]. Moreover, administration of FK866, a specific visfatin inhibitor, restored the phenotype [96]. Consistent with this, activation of the innate immune toll-like receptor 4 (TLR4) also induced visfatin expression in these cells [96]. Interestingly, both TLR4 and the IL1β receptor, known as interleukin 1 receptor (IL1R), share part of their downstream signaling pathway (Figure 3).

Apart from blocking IGF1 anabolic activity, as well as contributing to PGE2 synthesis and IL1β catabolic effects, visfatin induced other catabolic responses. It promoted the expression and release of a set of proteases, including ADAMTS4 and 5, as well as matrix metalloproteinases 3 (MMP3) and 13 (MMP13) in mouse chondrocytes [33]. Similar behavior was observed in porcine explants of cartilage where visfatin promoted MMPs’ activity, nitric oxide production, and proteoglycan release [109]. Moreover, in mouse chondrocytes, visfatin also reduced the production of aggrecan (ACAN) and high molecular weight proteoglycans [33]. In agreement to this, visfatin also reduced the mRNA expression of collagen type II (COL2A1) and type X (COL10A1), two structural proteins in the cartilage [110] (Figure 3). Additionally, visfatin has been related to other catabolic processes through its ability to up-regulate metallothionein 2 (*Mt2*), which is a metal homeostasis regulator that might be involved in OA development [111]. Nonetheless, since a chondroprotective role was also described for metallothionein 1 (*Mt1*) and *Mt2* [111], it is still unclear the specific effect of visfatin on these factors.

Visfatin activity has also been related to inflammatory activities. In mouse chondrocytes, the expression of monocyte chemoattractant protein 1 (*Mcp1*), interleukin 6 (*Il6*), and interleukin 8 (*Il8*) also known as chemokine (*Kc*) [42] were induced by visfatin (Figure 3).

It has been suggested that visfatin could also be involved in OA-associated pain, because it induced, in human and mouse chondrocytes, the increase of mRNA expression and release of nerve growth factor (NGF) [112] (Figure 3).

### 4.3. OA Epigenetics and Circadian Rhythm

Some actions of visfatin have been attributed to its link to sirtuin 1 (SIRT1) [96,99,105], the NAD^+^-dependent histone deacetylase, that is involved in the epigenetic regulation of multiple processes [51,52]. In fact, SIRT1 activity was reported to be enhanced by visfatin in rabbit and mice chondrocytes [96,99,105], but also, SIRT1 itself has been related to the induction of visfatin expression in human and rabbit chondrocytes [96,113]. Therefore, a positive feedback loop between visfatin and SIRT1 has been suggested [96] (Figure 3). Remarkably, this link has been associated with contradictory results in terms of chondrocyte catabolic and anabolic processes. Accordingly, it was determined that SIRT1 mediated the induction of visfatin and MMPs production in IL1β-stimulated human chondrocytes [113]. Likewise, up-regulation of visfatin and the consequent activation of SIRT family members were required to enhance MMPs’ expressions, and cartilage destruction in a mice OA model, induced by the overexpression of visfatin or the Hypoxia-inducible factor-2α (HIF2α) [105]. Additionally, the activation visfatin-SIRT1 axis in rabbit chondrocytes down-regulated the expression of SRY-box 9 (SOX9) and COL2, key factors involved in the maintenance of chondrocyte metabolism [96] (Figure 3).

Opposite to all the catabolic properties of visfatin described above, it was reported in human chondrocytes that visfatin, through SIRT1, exhibited certain anabolic effects, such as the maintenance of the expression of ACAN and COL2, two structural proteins of the cartilage [99] (Figure 3).

The alteration of the circadian rhythm in aged cartilage has been proposed as a connection between aging and OA [114]. In fact, two members of the molecular circadian clock, the circadian locomotor output cycles protein kaput (CLOCK), and the aryl hydrocarbon receptor nuclear translocator-like protein 1 (ARNTL), also called BMAL1, have been supposed to play a key role in OA development [114,115,116]. Interestingly, in mouse cartilage explants, visfatin gene expression was described to follow a circadian rhythm pattern [117]. In fact, *BMAL1* inhibition in cultured human chondrocytes involved the inhibition of visfatin expression, as well as *SIRT1* expression [102], which is a known regulator of the circadian system [118]. According to this, in different cell types, it was observed that the circadian clock through the regulation of visfatin expression, and therefore SIRT1 activity, formed a feedback regulatory loop [119,120]. As a result, it has been suggested that alterations of this loop in the aged cartilage might be involved in OA development [114].

### 4.4. Other OA Joint Tissues

Supporting the idea that OA is a disease of the whole joint, other tissues apart from the cartilage have been related to OA development. In this regard, it is noteworthy that the infrapatellar far pad (IFP) has been described to contribute to joint visfatin levels, and to OA joint inflammation [121,122]. Remarkably, OA patients secrete more visfatin from the IFP than from the subcutaneous adipose tissue [123]. Additionally, it was also determined that osteophytes, which are bone protuberances of the subchondral bone, are an important source of visfatin in the OA-joint [100].

However, the tissue that exhibited the highest level of visfatin expression was the synovium [42]. Supporting this fact, visfatin expression in OA synovial fibroblasts was highly induced upon stimulation with different inflammatory stimuli [124]. Accordingly, visfatin activity was identified ex vivo in the synovium [42]. Moreover, in human OA synovial fibroblasts, visfatin promoted the expression and synthesis of several inflammatory factors, like IL6 and TNFα [125].

Interestingly, meniscal degeneration may be an early event in knee OA that could be boosted by certain adipokines, including visfatin [109,126]. In fact, visfatin stimulation of porcine meniscus was able to increase nitric oxide production, MMP activity, and matrix degradation [109]. Consistent with this, it was described that meniscal cartilage was more susceptible to adipokines catabolic activities than the articular cartilage [126].

## 5. Visfatin’s Role in Osteoporosis

### 5.1. The OP and Visfatin Connection

Visfatin is highly transcribed in human bone marrow, which suggests its involvement in bone homeostasis [31]. In fact, certain genetic variations in this gene have been associated with an altered skeletal growth [127]. Considering that aging is a key factor for OP development, it is noteworthy that visfatin inhibition induced aging in the bone marrow-derived mesenchymal stem cells (BM-MSCs) of young rats, while its overexpression attenuated cell senescence in aged rat BM-MSCs [128]. Accordingly, visfatin has been widely studied in the context of bone physiology and pathology.

No association between BMD and visfatin circulating levels was observed in the metadata or other cohort-independent studies [129,130,131,132,133]. Besides, in another study, performed in postmenopausal women, visfatin serum levels were not significantly correlated with BMD after a multivariable regression analysis [134]. Similarly, the differences in BMD attributed to regular physical exercise were not associated with changes in visfatin serum levels [135].

### 5.2. Bone Catabolism and Inflammation

**Figure 4:**
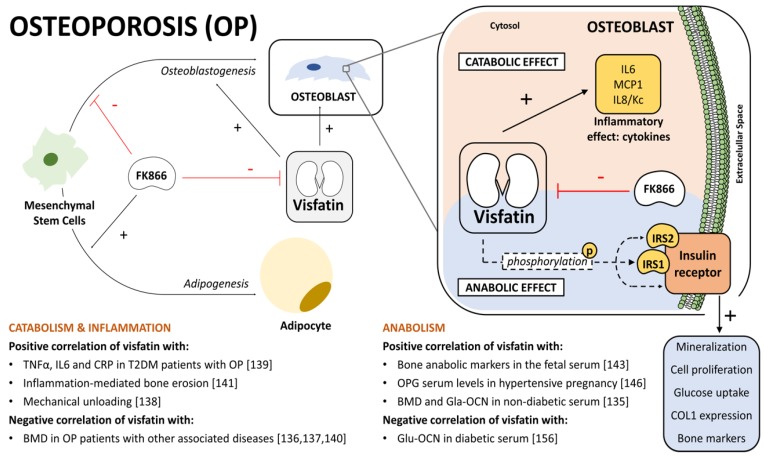
**Visfatin** Role in Osteoporosis (OP). Visfatin is involved in OP. Visfatin exhibits opposing effects on bone metabolism. It induces anabolic responses and also catabolic responses linked to inflammation and other diseases. Visfatin promotes the osteoblastogenesis process as well as anabolic effects on osteoblasts. Nonetheless, it also promotes certain inflammatory responses in these cells. Bone mineral density (BMD); carboxylated osteocalcin (Gla-OCN), uncarboxylated OCN (Glu-OCN); tumor necrosis factor α (TNFα); interleukin 6 (IL6); C-reactive protein (CRP); interleukin 8 (IL8), also known as chemokine (Kc); monocyte chemoattractant protein 1 (MCP1); insulin receptor substrates 1 (IRS1) and 2 (IRS2); collagen 1 (COL1).

Despite the above-mentioned lack of association between visfatin and BMD, in other studies, visfatin serum levels correlated negatively to BMD Z-scores at the lumbar spine and at the femoral neck in patients with an OP associated to a cardio-respiratory disease [136]. In Z-scores BMD were compared to the averaged BMD of patients with the same age and gender [136]. This negative correlation of visfatin serum levels and BMD was also observed in acromegalic patients [137]. Reinforcing the idea of an association between visfatin and bone catabolism, it was reported that visfatin serum levels were rapidly increased upon the exposition of healthy volunteers to mechanical unloading, a known pro-catabolic condition for the bone [138] (Figure 4).

Osteoporosis and visfatin have been tightly associated with inflammatory processes [4,37]. T2DM patients with a secondary OP exhibited increased serum visfatin levels and inflammatory factors such as TNFα, IL6, and CRP [139]. In line with this, the presence of osteoporosis and lower BMD in inflammatory bowel disease (IBD) patients was associated with an increase of visfatin serum levels [140]. This relationship among visfatin, and the enhanced inflammation and suppressed bone metabolism, was further supported by animal models of arthritis. In these models, visfatin promoted bone loss, and other catabolic and inflammatory responses [141], which were halted by the specific visfatin inhibitor FK866 [142]. In agreement with this, visfatin inhibition by FK866 diminished pro-inflammatory factors (*Il6*, *Il8/Kc*, and *Mcp1*) in mouse osteoblasts [42] (Figure 4).

### 5.3. Bone Anabolism

Bone anabolism has been associated with human serum levels of visfatin in a few reports [143,144,145,146]. These reports described a positive correlation between serum visfatin levels and BMD in healthy controls [144,145], as well as with osteoprotegerin (OPG) levels in hypertensive pregnant women [146]. Additionally, fetal circulating levels of visfatin were correlated with bone anabolic markers [143] (Figure 4).

Despite the limited number of reports linking visfatin circulating levels and bone anabolism, a significant bulk of evidence depicts a pro-anabolic role of visfatin in the context of osteoblast differentiation and function. Visfatin knock-down or inhibition in mouse BM-MSCs reduced the osteoblastogenesis of these cells, alkaline phosphatase (ALP) activity, matrix mineralization, and the expression of osteoblast differentiation markers [147] (Figure 4). Likewise, bone marrow stromal cells from visfatin^+/−^ mice, as well as different visfatin-deficient osteoblastogenic cell lines, exhibited diminished mineralization, expression, and activity of key osteoblastic markers, ALP and Runt-related transcription factor 2 (*Runx2*) [148]. The underlying mechanism of this visfatin-mediated promotion of osteoblast differentiation was partially explained by an epigenetic process that involved the modification of H3-Lys9 acetylation [148].

Osteoblast metabolism and glucose metabolism are mutually related [149]. According to this, it was suggested that some visfatin anabolic effects on the bone might be related to its insulin-mimetic activity [150]. In fact, tyrosine phosphorylation of the insulin receptor substrate 1 (IRS1) and 2 (IRS2), as well as the insulin receptor in human osteoblasts was induced by visfatin [150]. It also induced the proliferation of these cells, their glucose uptake, and collagen type I expression [150]. Interestingly, visfatin also induced human osteoblast matrix mineralization without modifying ALP activity [150]. Nonetheless, although visfatin down-regulated osteoblast-mediated osteocalcin (OCN) secretion [150], other authors reported that serum visfatin levels positively correlated to carboxylated OCN (Gla-OCN) in healthy subjects [135] (Figure 4). In agreement with these, visfatin inhibitor FK866 reduced the mineralization and increased the adipogenesis process of mouse bone marrow stromal cells [151]. Moreover, visfatin deletion or inhibition in the mouse mesenchymal cell line C3H10T1/2 inhibited osteoblastogenesis [152] and promoted adipogenesis [151]. The involvement of visfatin in osteoblast differentiation was further observed in the mouse pre-osteoblastic cell line MC3T3-E1 [152] (Figure 4). Interestingly, the differentiation to osteoblasts induced the expression of visfatin in both mouse cell lines, C3H10T1/2 and MC3T3-E1, as well as in BM-MSCs, which suggests the involvement of visfatin in the osteoblastogenesis process [147,152].

Reliable data on visfatin’s contribution to osteoclast differentiation is scarce and somewhat contradictory. It has been described in humans that visfatin’s inhibitor FK866 down-regulated nuclear factor-κB (NFκB) activity, along with osteoclast differentiation from precursors cells [153]. However, other reports showed that in mouse and human monocytes, visfatin suppressed the osteoclastogenesis mediated by the receptor activator of an NFκB ligand (RANK-L) [154,155]. This effect was attributed to its ability to inhibit *RANK* expression and signaling [154,155]. Supporting this relationship, it was found that type II diabetic patients had their visfatin levels in the serum negatively correlated with undercarboxylated OCN (Glu-OCN) which might be related to a reduced osteoclast-mediated decarboxylating activity [156].

## 6. Visfatin as a Potential Biomarker and Therapeutic Target

In the rheumatology field where musculoskeletal pathologies lie, the foremost objective is to find preventive tools to avoid the onset and progression of these diseases. To achieve that ultimate goal, the search for biomarkers is fundamental for early diagnosis and treatment. The research done so far on visfatin in the context of major MSP has shed light on the potential use of visfatin as a biomarker and therapeutic target.

The potential of visfatin for clinical diagnosis of OA relies on its established association with the disease progression [6,7,33,99,101,106,107]. The specific increase of visfatin expression in several OA joint tissues [32,92,93,94] suggests that its determination in the synovial fluid might be a useful early diagnostic tool for OA [100,101]. In fact, the tight relationship of visfatin synovial fluid levels with synovium inflammatory responses, cartilage degradation, and osteophyte formation may provide an accurate description of the catabolic and inflammatory processes taking place at incipient OA joints [100,101,103,104]. Nevertheless, it is noteworthy that certain OA comorbidities modulate visfatin’s circulating level, which may represent the biggest pitfall to overcome for its use as an OA-biomarker [9,12,13,14,15,16,17,18,73,74,75,76,77].

Visfatin is ubiquitously expressed in multiples tissues [9,12,31,32,33,34,35,36], hence the idea of its systemic inhibition to address OA local alterations might be not desirable. Instead, a local blockade of visfatin in the OA joint might be an appealing strategy, and henceforth research in this direction is needed [42,142].

Regarding OP, the negative correlation between visfatin serum levels and bone metabolism were found to be associated with systemic inflammation [4,37,139,140,141,142]. Despite the direct anabolic actions described for visfatin on osteoblast metabolism, its serum levels might be useful to identify inflammation-associated bone alterations [143,144,145,146,147,148,149,150]. This unclear dichotomy between anabolic and catabolic effects hampers any short-term use of visfatin as a diagnostic tool or therapeutic target for OP. Nonetheless, this might not preclude its clinical use. In fact, parathyroid hormone (PTH) also exhibits catabolic and anabolic effects on bone metabolism [157], and it is used as a therapeutic tool to treat bone loss in OP [158]. Consequentially, much research is needed in this front to determine the final use of visfatin on OP.

## 7. Conclusions

Visfatin is a ubiquitous life-essential enzyme with catabolic and inflammatory properties that are associated with osteoarthritis (OA) and osteoporosis (OP) shared risk factors.

In line with this, visfatin is also involved in OA joint pro-inflammatory and catabolic processes. Moreover, visfatin-Sirt1 axis works as a relevant epigenetic regulator of the OA cartilage. In the bone, visfatin exhibits opposing actions. It has been linked with inflammation-associated OP, while in osteoblasts it induces anabolic responses.

All in all, several pitfalls hamper the imminent use of visfatin as a biomarker of OA and OP. Similarly, the opposing effects of visfatin on bone metabolism present difficulties, but do not deter its future use as a therapeutic target. Interestingly, the potential use of visfatin as a therapeutic target stirs strong clinical interest in the local context of OA joints.
